# Antibody-Drug Conjugate Targeting c-Kit for the Treatment of Small Cell Lung Cancer

**DOI:** 10.3390/ijms23042264

**Published:** 2022-02-18

**Authors:** Kwang-Hyeok Kim, Jin-Ock Kim, Jeong-Yang Park, Min-Duk Seo, Sang Gyu Park

**Affiliations:** 1 College of Pharmacy, Ajou University, 206 World Cup-ro, Yeongtong-gu, Suwon-si 16499, Korea; kgh703@ajou.ac.kr (K.-H.K.); kjo8909@ajou.ac.kr (J.-O.K.); kiki_0409@naver.com (J.-Y.P.); mdseo@ajou.ac.kr (M.-D.S.); 2 Novelty Nobility, 227 Unjung-ro, Seongnam-si 13477, Korea

**Keywords:** c-Kit, small cell lung cancer, monoclonal antibody, antibody-drug conjugate

## Abstract

Lung cancer is the leading cause of cancer-related deaths. Small cell lung cancer (SCLC) accounts for 15–25% of all lung cancers. It exhibits a rapid doubling time and a high degree of invasiveness. Additionally, overexpression of c-Kit occurs in 70% of SCLC patients. In this study, we evaluated an antibody-drug conjugate (ADC) that targets c-Kit, which is a potential therapeutic agent for SCLC. First, we generated and characterized 4C9, a fully human antibody that targets c-Kit and specifically binds to SCLC cells expressing c-Kit with a binding affinity of K_D_ = 5.5 × 10^−9^ M. Then, we developed an ADC using DM1, a microtubule inhibitor, as a payload. 4C9-DM1 efficiently induced apoptosis in SCLC with an IC_50_ ranging from 158 pM to 4 nM. An in vivo assay using a xenograft mouse model revealed a tumor growth inhibition (TGI) rate of 45% (3 mg/kg) and 59% (5 mg/kg) for 4C9-DM1 alone. Combination treatment with 4C9-DM1 plus carboplatin/etoposide or lurbinectedin resulted in a TGI rate greater than 90% compared with the vehicle control. Taken together, these results indicate that 4C9-DM1 is a potential therapeutic agent for SCLC treatment.

## 1. Introduction

Lung cancer is the leading cause of cancer-related deaths in the Western world and is classified into two groups: small cell lung cancer (SCLC) and non-SCLC (NSCLC) [1]. SCLC, a neuroendocrine tumor, is distinguished from NSCLC by its rapid tumor growth, high degree of invasiveness and early development of widespread metastases [2]. SCLC is distinctly different from extrapulmonary small cell carcinoma with respect to disease progression, prognosis, and etiology [3]. Without proper treatment, the life expectancy of SCLC patients is less than four months. Although the five-year relative survival rate has improved by 7% over the last few decades, it remains extremely poor [4]. 

A variety of molecular markers have been implicated in the pathogenesis and prognosis of SCLC [5,6]. Paracrine or autocrine signal transduction pathways are widely used to explain dysregulated SCLC growth [6]. In addition, tumor protein p53, retinoblastoma protein, NOTCH, MYC, and phosphatidylinositol 3-kinase (PI3K) are aberrantly mutated in SCLC; however, well-established etiological factors, such as EGFR mutations that occur in NSCLC, have not been identified [7,8,9,10]. SCLC has a very aggressive course and is characterized by genomic instability, increased vascularity, and a high metastatic potential [11]. Consequently, most SCLC patients already present with metastatic disease outside of the chest, at the time of diagnosis, which results in premature death [12]. In addition, most SCLC patients are current or former heavy smokers, which is associated with a high tumor mutational burden, with C:G > A:T transversions being the most common type of base substitutions [13,14]

The c-Kit proto-oncogene encodes a transmembrane tyrosine kinase growth factor receptor that belongs to the platelet-derived growth factor receptor (PDGFR) family [15,16]. Its ligand stem cell factor (SCF) is a hematopoietic growth factor that promotes the proliferation of multiple hematopoietic stem cells [17,18]. In addition, c-Kit activity is dysregulated in various cancers [19,20]. Previous studies reported that the expression of c-Kit containing oncogenic mutations is either dysregulated and/or up-regulated in various cancers, which results in SCF-independent c-Kit activation and an aggressive form of cancer [20]. Interestingly, a variety of evidence indicates that SCLC cell lines and tumors express both the c-Kit receptor and SCF mRNA, suggesting that these gene products constitute an autocrine loop that mediates tumor cell survival and growth [21,22]. Although SCLC is significantly correlated with smoking, it does not contain oncogenic c-Kit mutations. Immunohistochemical staining showed that overexpression of c-Kit occurs in 70% of SCLC patients [23,24]. Imatinib, which was developed to target BCR-ABL, platelet-derived growth factor receptor (PDGFR), and c-Kit, is currently used to treat chronic myeloid leukemia, acute lymphoid leukemia, gastrointestinal stromal tumor (GIST), and hypereosinophilic syndrome [25,26,27,28]. A variety of in vitro and in vivo studies demonstrated that imatinib exhibits therapeutic efficacy against SCLC [29,30]. However, in phase 2 clinical trials, imatinib failed to exhibit significant therapeutic efficacy as shown by a lack of objective responses [31,32,33]. Thus, an alternative approach to target c-Kit in SCLC is needed. In this study, we generated and characterized 4C9, a human antibody targeting c-Kit. We developed an antibody-drug conjugate (ADC) using DM1, a microtubule inhibitor, coupled with N-succinimidyl-4-(N-maleimidomethyl) cyclohexane-1-carboxylate (SMCC) to generate 4C9-DM1, and then evaluated its therapeutic efficacy in vitro and in vivo.

## 2. Results

### 2.1. 4C9 Antibody Specifically Binds to c-Kit

**Figure 1:**
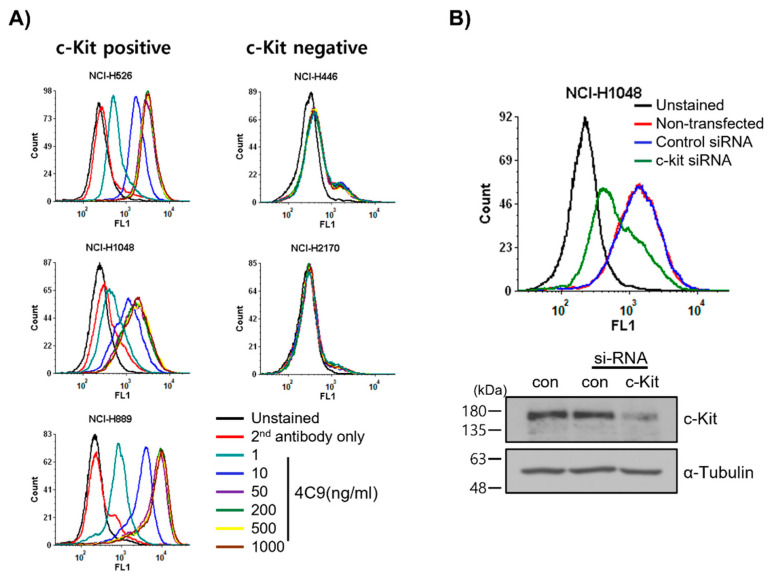
Determination of specific binding of the 4C9 antibody to the surface of SCLC cells. (**A**) SCLC cell lines were incubated with 4C9 antibody in a dose-dependent manner and analyzed by FACS. (**B**) NCI-H1048 cells were transfected with control or c-Kit si-RNA and incubated for 72 h. Binding of 4C9 (1 μg/mL) to NCI-H1048 cells was determined by FACS. The si-RNA-mediated down-regulation of c-Kit protein expression was evaluated by Western blot analysis. Alpha-tubulin was used as a loading control.

First, we examined whether the 4C9 antibody specifically binds to c-Kit on cell surface. FACS analysis revealed that 4C9 binds to various SCLC cell lines, including NCI-H526, NCI-H1048, and NCI-H889, in a dose-dependent manner (Figure 1A). Interestingly, 4C9 antibody binding was saturated at 50 ng/mL in c-Kit-positive SCLC cell lines. In addition, the expression of c-Kit was higher in NCI-H889 cells compared with that in NCI-H526 and NCI-H1048 cells, which is consistent with a previous report [34]. However, 4C9 did not show cross-reactivity with NCI-H446 and NCI-H2170 cells, which are c-Kit-negative SCLC cell lines. We further examined the specific binding of 4C9 to c-Kit using siRNA knockdown experiment. Western blot analysis showed that c-Kit siRNA efficiently decreased protein expression in NCI-H1048 cells (Figure 1B). FACS analysis demonstrated that c-Kit expression on the cell surface was also down-regulated by c-Kit siRNA. Taken together, 4C9 binds specifically to the extracellular domain of c-Kit on the cell surface.

**Figure 2:**
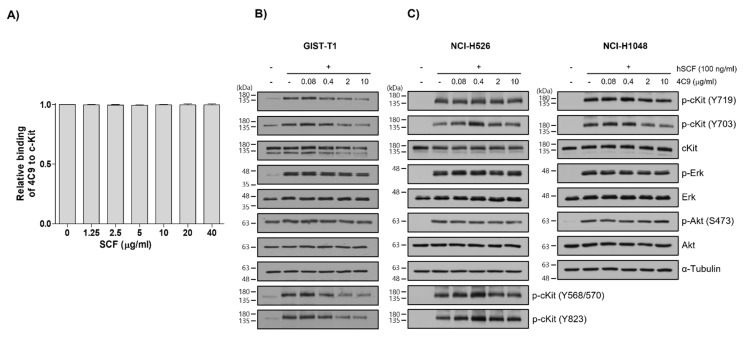
Characterization of the 4C9 antibody. (**A**) Human c-Kit (20 ng/well) was coated onto 96-well plates and the binding of the 4C9 antibody was investigated in the presence of human SCF at the indicated concentrations. The results represent the mean ± SD of three independent experiments. (**B**,**C**) GIST-T1, NCI-H526, or NCI-H1048 cells were treated with 4C9 at the indicated concentrations in the presence or absence of SCF (100 ng/mL). The phosphorylation of c-Kit, Akt, and ERK was assessed by Western blot analysis. In NCI-H1048 cells, phosphorylation of Y568/570 and Y823 by SCF treatment was not detected. Alpha-tubulin was used as a loading control. The results represent the mean ± SD of three independent experiments.

Next, we investigated whether the 4C9 antibody could inhibit the binding of SCF, a ligand for c-Kit. Competitive enzyme-linked immunosorbent assay (ELISA) results showed that the binding of 4C9 antibody to c-Kit was not affected by SCF, even at high concentrations (Figure 2A), suggesting that 4C9 antibody binds to c-Kit independent of SCF. In addition, we assessed whether 4C9 could inhibit SCF-mediated phosphorylation of c-Kit. Using GIST-T1 cells, pretreatment with 4C9 antibody resulted in decreased c-Kit phosphorylation in a dose-dependent manner; however, the phosphorylation levels of the ERK and Akt, downstream molecules of c-Kit, were not changed by treatment with 4C9 antibody (Figure 2B). Interestingly, total c-Kit levels decreased by 4C9 antibody treatment (Figure 2B). Hence, a decrease in phosphorylated c-Kit levels may result from decreased expression of c-Kit. A stability assessment of c-Kit indicated that the 4C9 antibody dramatically decreased total c-Kit levels in a time-dependent manner in both GIST cell lines and in some SCLC cell lines (Supplementary Figure S1), which may be associated with ubiquitination-dependent degradation. Nevertheless, this needs further elucidation. In contrast to GIST-T1, 4C9 did not reduce SCF-mediated c-Kit phosphorylation or c-Kit stability in the NCI-H526 and NCI-H1048 cell lines (Figure 2C). Furthermore, the 4C9 antibody did not inhibit phosphorylation of ERK and Akt induced by SCF, which suggests that the 4C9 antibody does not function as an antagonist of SCF/c-Kit signaling.

### 2.2. Generation and Characterization of ADC (4C9-DM1)

**Figure 3:**
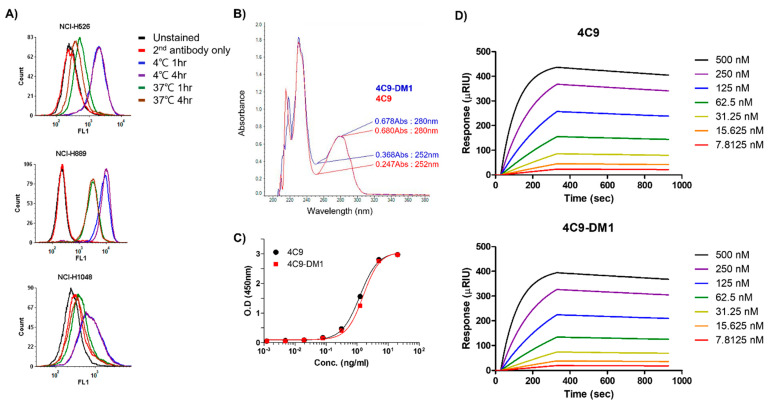
Characterization of 4C9-DM1. (**A**) The internalization of the 4C9 antibody into various SCLC cell lines was determined using FACS analysis. SCLC cells were incubated with cycloheximide (75 μg/mL) and blocked with Fc blocker for 10 min to inhibit Fc receptor-mediated internalization. SCLC cells were incubated in the presence or absence of the 4C9 antibody at 4 °C or 37 °C for 1–4 h and subjected to flow cytometry. The fluorescent signal of the 4C9/c-Kit complex on the cell surface decreased after incubation at 37 °C. (**B**) Optical absorbance of 4C9-DM1 compared with that of the naked 4C9 antibody at 252 nm. The binding affinity of 4C9 and 4C9-DM1 to c-Kit was compared using ELISA (**C**) and SPR (**D**).

Even though c-Kit is overexpressed in SCLC cell lines, naked antibodies cannot be applied to treat SCLC because of the limited contribution of SCF/c-Kit signaling in the pathogenesis of SCLC and failure of imatinib [32,33]. Therefore, the development of an ADC would be a reasonable option to effectively treat SCLC. One important factor to consider when creating an ADC is the internalization efficiency after the complex formation of the antibody with the target molecule. Therefore, we first investigated whether the 4C9 antibody is internalized in SCLC cell lines. FACS analysis exhibited that the internalization efficiency of the 4C9 antibody was 91% in NCI-H526, 76.6% in NCI-H1048, and 68.6% in NCI-H889 cells (Figure 3A), which suggests that the 4C9 antibody could be used as an efficient carrier for the specific delivery of toxin to treat SCLC. Next, we generated an ADC using SMCC-DM1, which consists of a noncleavable linker and a microtubule inhibitor. Because DM1 absorbs ultraviolet light at 252 nm [35], the absorbance of the naked antibody (4C9) and ADC (4C9-DM1) at 252 nm was analyzed. The absorbance of 4C9-DM1 was higher than that of 4C9 at 252 nm (Figure 3B). The drug-antibody ratio (DAR) was determined as described previously [36] and the DAR was approximately 2.16. SDS-PAGE analysis exhibited that the conjugation of SMCC-DM1 to the 4C9 antibody resulted in a slight size shift (Supplementary Figure S2). Since N-hydroxysuccinimide ester of the SMCC linker reacts with primary amines of lysine residues in the antibody, the target binding affinity of ADC may be affected. An ELISA demonstrated that the binding affinities of 4C9 and 4C9-DM1 to c-Kit were similar (Figure 3C). A quantitative analysis of the binding affinity using surface plasmon resonance (SPR) indicated that the binding affinity of 4C9 to human c-Kit was 5.5 × 10^−9^ M (Ka = 2.27 × 10^4^ M^−1^s^−1^ and Kd = 1.27 × 10^−4^ s^−1^), and 4C9-DM1 also showed similar binding affinity (5.46 × 10^−9^ M; Ka = 2.14 × 10^4^ M^−1^s^−1^ and Kd = 1.16 × 10^−4^ s^−1^) (Figure 3D). Taken together, these results indicate that conjugation using SMCC-DM1 to the 4C9 antibody did not affect the binding affinity of 4C9 antibody for c-Kit.

### 2.3. 4C9-DM1 Exhibits Antitumor Activity In Vitro and In Vivo

**Figure 4:**
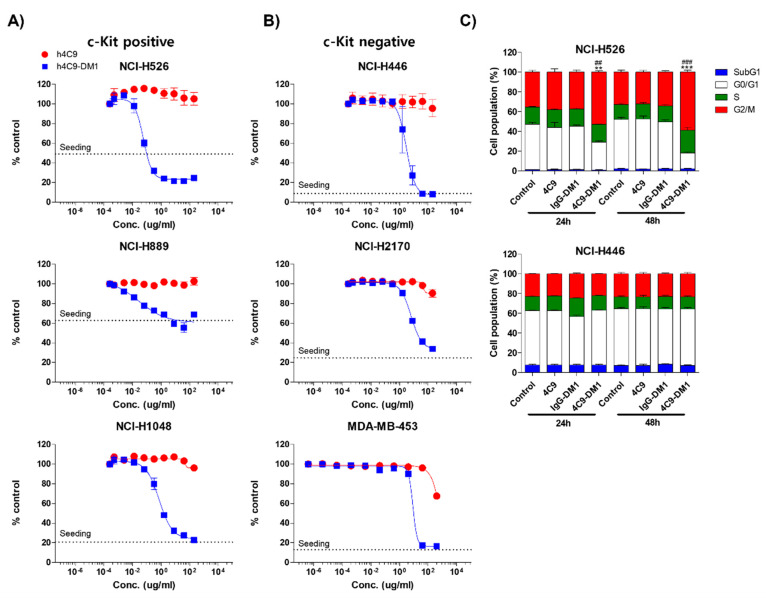
4C9-DM1 exhibits cytotoxicity against SCLC cells in vitro. (**A**,**B**) c-Kit positive or negative SCLC cell lines were seeded into 96-well plates and incubated with 4C9 or 4C9-DM1 in a dose-dependent manner for 3–5 days. Live cells were stained with Hoechst 33342 (10 μM) at 37 °C for 30 min and quantitated using a Celigo Imaging Cytometer. Treatment with 4C9-DM1 decreased cell viability in a dose-dependent manner. The results represent the mean ± standard error of the mean of at least three independent experiments. (**C**) 4C9-DM1 induced cell cycle arrest at the G2/M phase. SCLC cell lines were seeded into 96-well plates and incubated with 4C9 (1 μg/mL), IgG-DM1 (1 μg/mL), or 4C9-DM1 (1 μg/mL) for 24 and 48 h. Then, the cells were stained with propidium iodide and analyzed using a Celigo Imaging Cytometer (** and *** vs. control; ^##^ and ^###^ vs. IgG-DM1). The results represent the mean ± standard error of the mean of at least three independent experiments. The results are presented as the mean ± standard error of the mean. The means were compared using an unpaired Student’s two-sided *t*-test. ** *p* < 0.01, *** *p* < 0.001, ^##^ *p* < 0.01, ^###^ *p* < 0.001.

Next, we analyzed the in vitro cytotoxicity using various SCLC cell lines (NCI-H526, NCI-H889, NCI-H1048, NCI-H446, and NCI-H2170) and a breast cancer cell line (MDA-MB-453). The c-Kit negative cell lines (NCI-H446, NCI-H2170, and MDA-MB-453) were used as negative controls. 4C9-DM1 exhibited in vitro cytotoxicity against NCI-H526, NCI-H889, and NCI-H1048 with half-maximal inhibitory concentration (IC_50_) values ranging from 158 pM to 4 nM. The in vitro cytotoxic activity of 4C9-DM1 against c-Kit-positive cancer cell lines was 4- to >300-fold higher than that against c-Kit-negative cancer cell lines (Figure 4A,B and Table 1). Interestingly, the cytotoxic activity of DM1 against the c-Kit-positive cancer cells was 7- to >77 fold higher when applied as ADC rather than as payload alone. By contrast, its cytotoxic activity was 2–5 times lower against c-Kit-negative cancer cells (Table 1), suggesting the inclusion of a payload as an ADC may reduce off-target toxicity. DM1 induces cell cycle arrest at the G2/M phase by inhibiting the assembly of microtubules, resulting in apoptosis of actively dividing cells. Therefore, we analyzed the effect of 4C9-DM1 on the cell cycle using NCI-H526, a c-Kit positive SCLC cell line, and NCI-H446, a c-Kit negative SCLC cell line. As shown in Figure 4C, 4C9-DM1 significantly increased the cell population in the G2/M phase in a time-dependent manner but 4C9 and IgG-DM1 did not. In addition, 4C9, 4C9-DM1, and IgG-DM1 did not alter the cell population in the G2/M phase in c-Kit negative NCI-H446 cells, suggesting that cell cycle arrest in NCI-H526 cells is specifically mediated by DM1 delivered by complex formation with 4C9-DM1 and c-Kit.

**Figure 5:**
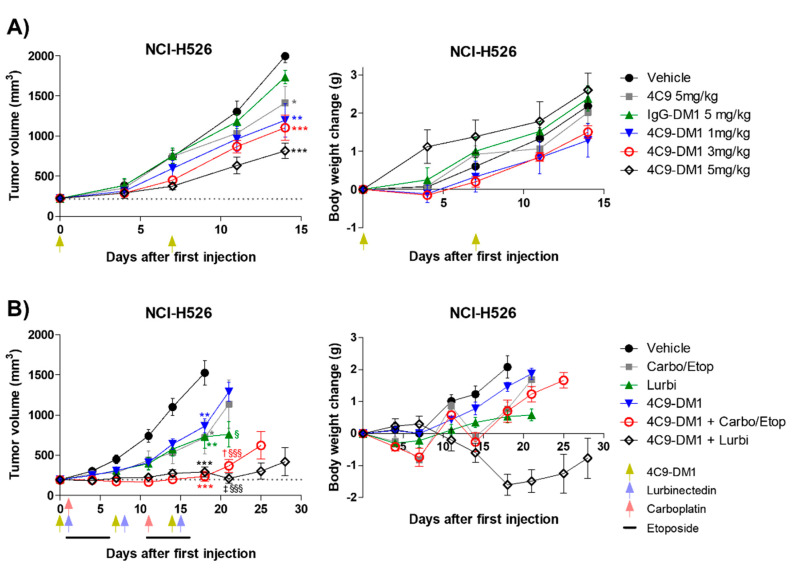
4C9−DM1 suppresses SCLC tumor growth in a xenograft mouse model. (**A**,**B**) Antitumor activity of 4C9-DM1 was evaluated in an in vivo xenograft mouse model. NCI-H526 cancer cells were implanted into immune-deficient mice as described in the Methods section. Mice with established tumors were randomized into different treatment groups when the tumor volume reached ~200 mm^3^ (*n* = 6). The animals were intravenously administered vehicle, 4C9, IgG-DM1, or 4C9-DM1. Carboplatin (60 mg/kg on days 1 and 11) and etoposide (3 mg/kg on days 1–5 and days 11–15) were intraperitoneally administered or combined with 4C9-DM1. Additionally, lurbinectedin (0.08 mg/kg on days 1, 8, and 15) was intravenously administered or combined with 4C9-DM1 as indicated. Green arrows indicate the administration of vehicle, IgG-DM1, 4C9, or 4C9-DM1, and blue and red arrows indicate the administration of lurbinectedin and carboplatin, respectively (*, **, and *** vs. their respective corresponding vehicle control; ^§^ and ^§§§^ vs. their respective corresponding 4C9-DM1 control; ^†^ vs. carboplatin/etoposide; ^‡^ vs. lurbinectedin). The results are presented as the mean ± standard error of the mean. The means were compared using an unpaired Student’s two-sided *t*-test. * *p* < 0.05, ** *p* < 0.01, *** *p* < 0.001, ^†^ *p* < 0.05, ^‡^ *p* < 0.01, ^§^ *p* < 0.05, ^§§§^ *p* < 0.001.

Based on the in vitro cytotoxicity assay, the in vivo efficacy of 4C9-DM1 was examined using mouse models xenotransplanted with NCI-H526. Although IgG-DM1 did not exert an effect, 4C9-DM1 significantly suppressed NCI-H526 tumor growth in a dose-dependent manner (Figure 5A and Supplementary Figure S3A). Tumor growth inhibition (TGI) rates of 4C9-DM1 at doses of 1, 3, and 5 mg/kg were 40%, 45%, and 59%, respectively, compared with that of the vehicle control. The 4C9 antibody alone partially inhibited tumor growth, but this effect was not statistically significant. Body weight losses due to the administered materials were not observed (Figure 5A and Supplementary Figure S3B), suggesting that there was no concern related to toxicity. Chemotherapy, including etoposide, cisplatin, carboplatin, and lurbinectedin are used to treat SCLC patients as the standard of care [37,38]. Although combination therapy using chemotherapeutic drugs is effective, most SCLCs rapidly recur within 1 year [39]. Therefore, we determined whether the combination of 4C9-DM1 with chemotherapy could enhance the therapeutic efficacy of SCLC. As shown in Figure 5B and Supplementary Figure S3C, 4C9-DM1, lurbinectedin, and carboplatin/etoposide exhibited similar antitumor activities; the TGI rates of these groups were 50% at day 18, compared with that of the vehicle control. Interestingly, the combination of 4C9-DM1 with lurbinectedin or carboplatin/etoposide synergistically suppressed tumor growth. The TGI rates of both groups were 85%, compared with that of the vehicle control at day 18 with subsequent regrowth. The combinatorial treatment with 4C9-DM1 plus lurbinectedin induced body weight loss of approximately 10%; however, body weight increased again after cessation of the treatment (Figure 5B and Supplementary Figure S3D). This suggests that this combination may be used to treat SCLC with manageable or mild toxicity.

## 3. Discussion

Since antibodies are used as carriers for toxic payloads to treat cancer, specific binding to the target is critical to reduce off-target adverse events. Therefore, we investigated whether the 4C9 antibody exhibits off-target protein binding. The results of protoarray analysis using a chip embedded with >20,000 human proteins showed that the 4C9 antibody bound to protein phosphatase 1 regulatory subunit 3B (Ppp1r3b) (results not shown). Ppp1r3b is localized to intracellular membrane-bound granules in the liver and skeletal muscle, where it regulates energy homeostasis through glycogen synthesis [40]. This suggests that 4C9 does not bind to other extracellular proteins. Therefore, the 4C9 antibody can be efficiently used as a carrier of toxic payloads for treating cancer without significant off-target binding, at least in part.

SCF binding to c-Kit is mediated by electrostatic interactions of the charged residues in domain 2, as well as hydrogen bonds formed in domain 3 [41]. A competitive ELISA showed that 4C9 did not inhibit SCF binding to c-Kit (Figure 2A). In addition, the 4C9 antibody did not interrupt SCF-mediated phosphorylation of c-Kit (Figure 2B,C), suggesting that the binding site of the 4C9 antibody to c-Kit is different from the SCF binding site. In this study, phosphorylation of c-Kit decreased by 4C9 antibody in GIST cell lines, but not in SCLC cell lines (Figure 2C), which is mediated by decreased c-Kit stability. c-Kit expression is negatively regulated by the ubiquitin E3 ligase, including c-casitas B-cell lymphoma (c-Cbl) and suppressor of cytokine signaling 6 [42,43,44]. The different stability observed for c-Kit following treatment with the 4C9 antibody in GIST and SCLC cell lines may result from varying expression or activity of E3 ligases, which needs further elucidation.

SCF/c-Kit signaling induces activation of various signaling mediators, including PI3K/Akt/mammalian target of rapamycin (mTOR) pathway and the mitogen-activated protein kinase kinase/extracellular signal-regulated kinase (MEK/ERK) pathway in SCLC and was inhibited by imatinib [21,30]. Interestingly, however, when SCLC cells are plated on laminin, resistance to apoptosis is induced by imatinib because of the laminin-mediated increased activation of the mTOR pathway [45], which may contribute to the failure of phase 2 clinical trial [31,32,33]. In the present study, we further confirmed that although various c-Kit wild-type SCLC cell lines, including NCI-H526, NCI-H889, and NCI-H1048, were treated with imatinib up to 10 μM, >90% cell viability was maintained, whereas the IC_50_ of imatinib was 0.03–0.3 μM in c-Kit-positive GIST cells in which imatinib is the standard of care (Supplementary Figure S4 and Supplementary Table S1). Therefore, there is a need for novel therapeutics for c-Kit-targeted therapy to treat SCLC. As shown in Figure 4 and Figure 5, the ADC targeting c-Kit, represents an alternative treatment. Although chemotherapy in SCLC clinics shows good response rates, most SCLCs rapidly recur within 1 year and result in death [39]. After DNA damage, various proteins, including ATM, ATR, DNA-PK, and Rad5 are imported into the nucleus on interphase microtubules to repair damaged DNA. The trafficking of proteins for DNA repair is interrupted by microtubule inhibitors, leading to increased sensitivity to DNA damaging agents [46]. The combination of paclitaxel, Docetaxel, or vinorelbine with cisplatin or carboplatin for SCLC treatment and the combination of cyclophosphamide or capecitabine with Docetaxel for breast cancer treatment are routinely administered to patients [46]. 4C9-DM1 induces cell cycle arrest by inhibiting microtubule assembly, leading to cancer cell apoptosis. In addition, lurbinectedin covalently binds to the minor groove of the DNA duplex, resulting in double-strand DNA breaks. Furthermore, lurbinectedin inhibits RNA-polymerase II activity and promotes its ubiquitin-mediated proteasomal degradation [47]. Thus, we hypothesized that combination therapy with carboplatin/etoposide or lurbinectedin may increase therapeutic efficacy. As shown in Figure 5, combination therapy synergistically decreased tumor volume in mouse xenograft model. Since our study showed that lurbinectedin exhibits a higher toxicity than carboplatin/etoposide, the combination of 4C9-DM1 with carboplatin/etoposide would be the preferred treatment regimen.

Atezolizumab and durvalumab targeting PD-L1 are being used in combination with chemotherapy [48,49]. Nevertheless, the responses are limited because the expression of PD-L1 occurs in only 22–26% of SCLC tumors and in 18% of tumor-infiltrating macrophages in patients [50,51]. Since overexpression of c-Kit has been reported in 70% of SCLC patients [23,24], determination of c-Kit and PD-L1 expression using immunohistochemical staining in the tissues of SCLC patients may increase the therapeutic efficacy by combination treatment with anti-c-Kit ADC plus anti-PD-L1. In summary, we developed a fully human antibody targeting c-Kit, characterized its binding specificity, and examined antitumor activity using various in vitro and in vivo models for application as an ADC. Our results indicate that 4C9-DM1 is a potential therapeutic ADC that can be used to treat SCLC. 

## 4. Materials and Methods

### 4.1. Generation and Production of 4C9 Antibody

A fully human 4C9 antibody targeting human c-Kit was produced as described previously [52]. Briefly, a human recombinant c-kit (Q26-T520, Elabscience, Wuhan, China) was immunized into humanized NSG mice (Orient Bio, Sungnam, Korea) implanted with human CD34^+^ hematopoietic stem cells (Lonza, Basel, Switzerland). The emulsion was produced by mixing the c-kit protein (1 μg/μL) with an equal volume of complete Freund’s adjuvant (Sigma-Aldrich, St. Louis, MO, USA). Booster injections were administered during week 5. The human antibody titer in mouse serum was assessed using an indirect ELISA. Maxisorp plates were coated with the c-kit protein at 0.1 μg/well. Mice with a positive immune response were subjected to a final boost injection in week 7. Hybridomas with positive reactivity against c-kit in the ELISA were subcloned using a standard limiting dilution method. The antibody gene sequences of the heavy and light chain variable domains were determined by GenScript (Piscataway, NJ, USA). The nucleotide sequence of the 4C9 clone was codon optimized for *Cricetulus griseus*, synthesized as IgG1, and subcloned into the pCHO1.0 vector (Thermo Fisher Scientific, Waltham, MA, USA). The recombinant plasmids were transiently transfected into CHO-S cells cultured in CD CHO serum-free medium using the ExpiCHO™ Expression System Kit (Thermo Fisher Scientific, Waltham, MA, USA) for expression of the 4C9 antibody. The antibody was purified using Protein A Sepharose and SP Sepharose columns (Invitrogen, Carlsbad, CA, USA) as described previously [52].

### 4.2. Cell Lines and Culture

The GIST cell line (GIST-T1) was kindly provided by Dr. Sebastian Bauer (University of Duisburg-Essen, Duisburg, Germany). SCLC cell lines (NCI-H446, NCI-H526, NCI-H889, NCI-H1048, and NCI-H2170) and a breast cancer cell line (MDA-MB-453) were purchased from ATCC (Manassas, VA, USA). All cell lines were cultured according to the manufacturer’s instructions and maintained at 37 °C in humidified 5% CO_2_ incubator.

### 4.3. Flow Cytometry

To determine the binding of 4C9 to c-Kit on the cell surface, flow cytometry was performed. The proteins on the cell surface (2.0 × 10^5^) were blocked with 1 × DPBS containing 5% BSA on ice for 1 h. The cells were then incubated with the 4C9 antibody at the indicated concentrations on ice for 1 h. After washing three times with 1 × DPBS containing 2% BSA, the cells were stained with 0.3 μg/mL FITC-conjugated anti-human IgG secondary antibody (Invitrogen, Carlsbad, CA, USA) on ice for 1 h. Fluorescence was detected using a CyFlow Cube6 (Sysmex Partec, Goerlitz, Germany).

### 4.4. Western Blot Analysis

For SCF/c-Kit signaling analysis, the cells were serum starved for 30 min and then pretreated with 4C9 antibody at the indicated concentrations for 30 min. Next, 100 ng/mL recombinant human SCF (R&D Systems, Minneapolis, MN, USA) was added and incubated for an additional 5 min. The cells were washed twice with DPBS and lysed using RIPA buffer (20 mM Tris-HCl, pH 7.6, 150 mM NaCl, 1 mM Na_2_EDTA, 1 mM EGTA, 1% NP-40, 1% sodium deoxycholate, 0.1% SDS, 10 mM β-glycerophosphate, 1 mM Na3OV4, 10 mM NaF, 1 μg/mL leupeptin, 1 mM PMSF, 5 μg/mL aprotinin, and 2 mM 2-mercaptoethanol). The protein extracts were subjected to SDS-PAGE and transferred onto PVDF membranes (Millipore, Burlington, MA, USA). The membranes were blocked with 1 × TBST containing 5% BSA at room temperature (RT) for 1 h and probed with the following antibodies: anti-c-Kit (R&D Systems, Minneapolis, MN, USA), anti-p-c-Kit (Tyr568/570, Tyr703, Tyr719, and Tyr823; Cell Signaling Technology, Beverly, MA, USA), anti-Erk1/2 (Santa Cruz Biotechnology, Santa Cruz, CA, USA), anti-p-Erk1/2 (Cell Signaling Technology, Beverly, MA, USA), anti-Akt (Santa Cruz Biotechnology, Santa Cruz, CA, USA), anti-p-Akt (Ser473; Cell Signaling Technology, Beverly, MA, USA), and anti-α-tubulin (generated in our laboratory).

### 4.5. si-RNA Study

For the siRNA study, cells were seeded into 150 mm^2^ plates. After 24 h, the culture medium was replaced with an Opti-MEM medium (Gibco, Billings, MT, USA). The cells were then transfected with control siRNA or c-Kit siRNA (200 pmol; Santa Cruz Biotechnology, Santa Cruz, CA, USA) using Lipofectamine RNAiMAX reagent (Invitrogen, Waltham, MA, USA) for 72 h according to the manufacturer’s instructions. c-Kit knockdown was confirmed by flow cytometry and Western blot analysis.

### 4.6. ELISA

To determine whether the 4C9 antibody interferes with SCF binding to c-Kit, a competitive ELISA was performed. Recombinant human c-Kit protein (20 ng/well; R&D Systems, Minneapolis, MN, USA) was coated onto a 96-well plate at 4 °C overnight. Each well was blocked with 1 × PBS containing 5% BSA at RT for 1 h. The human SCF protein was added to c-Kit protein-coated 96-well plates in a dose-dependent manner for 10 min. 4C9 antibody (2 ng/mL) was then added to each well at RT for 1 h. The plate was washed with 1 × PBS containing 0.1% Tween-20 four times and incubated with HRP-conjugated anti-human IgG secondary antibody (1:2000; Santa Cruz Biotechnology, Santa Cruz, CA, USA) for 1 h. Then, 3, 3′, 5, 5′-tetramethylbenzidine solution (Thermo Fisher Scientific, Waltham, MA, USA) was added for 5 min. The signal was measured at 450 nm using a SPECTROstar Nano Microplate Reader (BMG LABTECH, Ortenberg, GermanyELISA was also used to compare the binding affinities of 4C9 and 4C9-DM1 as previously described [34].

### 4.7. Internalization Assay

The internalization assay was carried out as previously described [34]. Briefly, the cells were incubated with cycloheximide (75 µg/mL) to prevent the transport of de novo synthesized c-Kit to the cytoplasmic membrane. Then, the cells were blocked with human BD Fc block™ (BD Biosciences, San Diego, CA, USA), to inhibit the Fc receptor-mediated internalization of antibodies, and 5% BSA (CellNest, Gyeonggi-do, Korea) at RT for 10 min. The cells were washed with PBS and incubated in the presence or absence of the 4C9 antibody (1 µg/mL) at 4 °C or 37 °C for 1–4 h. The cells were then incubated with FITC-conjugated anti-human IgG (0.3 μg/mL, Invitrogen) for 1 h, washed three times with PBS, and analyzed using a CyFlow^®^ Cube 6 (PARTEC).

### 4.8. Generation of ADC

First, the 4C9 antibody and normal human IgG1(Sino Biological, Beijing, China) were dialyzed with conjugation buffer (0.1 M sodium phosphate, 0.15 M sodium chloride, pH 7.2). The antibodies and SMCC-DM1 (MedChemExpress, Monmouth Junction, NJ, USA), linker-payload conjugates, were mixed in a 1:5 molar ratio, and the mixture was rotated at RT for 90 min. To remove the aggregated ADC, the products were centrifuged at 20,000× *g* and filtered through a Supor (Hydrophilic polyethersulfone) membrane (0.2 μm, Pall Life Sciences, Port Washington, NY, USA). ADC was purified with size exclusion chromatography (Superdex^TM^ 200, GE Healthcare, Wauwatosa, WI, USA) and dialyzed against storage buffer (10 mM sodium succinate, 0.05% polysorbate 20, and 6% sucrose; pH 5.0). The average drug-antibody ratio (DAR) of 4C9-DM1 was determined by comparing the absorbance of the naked antibody and ADC at 252 nm. In addition, the molar concentration of the linked DM1 was assessed by UV spectroscopy by measuring the different absorbance maxima of 4C9 and SMCC-DM1 at 280 and 252 nm, respectively, as reported previously [36]:

*A*280 = εAb280 × C_Ab_ + εD280 × C_D_ (1)

*A*252 = εD252 × C_D_ + εAb252 × C_Ab_ (2)

where:

εD280 = molar extinction coefficient of SMCC-DM1 at 280 nm.
εAb280 = molar extinction coefficient of 4C9 at 280 nm.
εD252 = molar extinction coefficient of SMCC-DM1 at 252 nm.
εAb252 = molar extinction coefficient of 4C9 at 252 nm.
C_D_ = molar concentration of SMCC-DM1.
C_Ab_ = molar concentration of 4C9.

Dividing Equation (2) by Equation (1) and after rearranging, Equation (3) was obtained.

DAR = C_D_/C_Ab_ = (εAb252 − RεAb280)/(RεD280 − εD252) (3)

where R is the total absorbance ratio (A252/A280). An optical path length of 10 mm was assumed using these equations. The molar extinction coefficients of SMCC-DM1 (εD280 = 5065 M^−1^ cm^−1^ and εD252 = 21,168 M^−1^ cm^−1^) were measured experimentally in our laboratory. For 4C9, the extinction coefficient values were as follows: εAb280 = 223,400 M^−1^ cm^−1^, as estimated, and εAb252 = 77,117 M^−1^ cm^−1^, as measured experimentally in our laboratories.

The spectra were recorded using a Nanodrop 2000 spectrophotometer (Thermo Fisher Scientific, Waltham, MA, USA). Samples were diluted to approximately 4.5 mg/mL in formulation buffer. A blank was recorded using the same buffer and measurements were performed at ambient temperature.

### 4.9. In Vitro Cytotoxicity Test

Cells (3–7 × 10^3^) were seeded into 96-well plates and incubated with 4C9 or 4C9-DM1 at the indicated concentrations for 3–5 days, on the basis of the growth rate of each cell line. Then the total cells were stained with 10 μM Hoechst 33342 (Thermo Fisher Scientific, Waltham, MA, USA) for 30 min and counted using a Celigo imaging cytometer (Nexcelom, Lawrence, MA, USA). For the cell cycle assay, 7–10 × 10^3^ cells were seeded into 96-well plates and incubated with 1 μg/mL 4C9, 4C9-DM1, or IgG1-DM1 for 24 and 48 h. Cells were fixed with 2% paraformaldehyde at 4 °C for 10 min (for suspension cell lines) or 80% cold ethanol at 4 °C for at least 2 h (for adherent cell lines). After washing twice with DPBS, cells were stained with 50 μg/mL propidium iodide solution (Sigma-Aldrich, St. Louis, MO, USA; containing 0.1 mg/mL RNase A, 0.05% Triton X-100) at 37 °C for 90 min. Fluorescence was analyzed using a Celigo imaging cytometer.

### 4.10. In Vivo Studies Using Xenograft Mouse Model

All animal studies were approved by the Ajou University Animal Care and Use Committee (IACUC 2019-0005), and all experiments were performed under the appropriate regulations and guidelines. NCI-H526 (2 × 10^6^ cells) or NCI-H1048 (5 × 10^6^ cells) cells with 50% Matrigel (Corning, NY, USA) were subcutaneously injected into the right flank of 5-week-old female C. B-17 mice (Orientbio, Seongnam, Korea). When the tumor volume reached ~200 mm^3^, drug administration was initiated. Naked antibody and ADC were intravenously administered at the indicated concentrations on days 0, 7, and 14. Lurbinectedin (0.09 mg/kg; MedChemExpress, Monmouth Junction, NJ, USA) was intravenously administered the day after ADC injection (days 1, 8, and 15). Carboplatin (60 mg/kg; Sigma-Aldrich, St. Louis, MO, USA) was administered intraperitoneally on days 1 and 11. Etoposide (3 mg/kg; Sigma, Sigma-Aldrich, St. Louis, MO, USA) was intraperitoneally administered on days 1–5 and 11–15. Tumor volumes were measured twice a week using a Vernier caliper, and the volume calculation formula was as follows: tumor volume = (4/3) × π × (length/2) × (width/2) × (height/2).

## Figures and Tables

**Table 1:** IC_50_ (nM) values of the tested materials.

c-Kit Expression	Tissue Type	Cell Line	4C9-DM1 *	SMCC-DM1
c-Kit positive	SCLC	NCI-H526	0.158	12.23
		NCI-H889	0.323	11.62
		NCI-H1048	4.08	30.45
c-Kit negative	SCLC	NCI-H446	16.58	6.97
		NCI-H2170	35.5	20.17
	Breast cancer	MDA-MB-453	47.63	9.763

* Molar concentration was calculated as 180 kDa for the molecular weight of 4C9-DM1.

## Data Availability

The data reported in this study are available from the corresponding author (sgpark@ajou.ac.kr) upon reasonable request.

## Supplementary Materials

The following supporting information can be downloaded at: https://www.mdpi.com/article/10.3390/ijms23042264/s1.

## Abbreviations

SCLC	Small cell lung cancer
ADC	Antibody-drug conjugate
TGI	Tumor growth inhibition
NSCLC	Non-small cell lung cancer
EGFR	Epidermal growth factor receptor
mTOR	Mammalian target of rapamycin
PI3K	Phosphoinositide 3-kinase
SCF	Stem cell factor
GIST	Gastrointestinal stromal tumor
SMCC	N-succinimidyl-4-(N-maleimidomethyl) cyclohexane-1-carboxylate
DAR	Drug-antibody ratio
IC50	Half-maximum inhibitory concentration
Ppp1r3b	Protein phosphatase 1 regulatory subunit 3B
PD-L1	Programmed death-ligand 1
