# Supplementary material for: Antibody-Drug Conjugate Targeting c-Kit for the Treatment of Small Cell Lung Cancer
Source: Int J Mol Sci. 2022 Feb 18;23(4):2264. doi: 10.3390/ijms23042264 (PMC8875948; doi:10.3390/ijms23042264)
Supplement: Supplementary file 1 [file ijms-23-02264-s001.zip › ijms-1593669-supplementary.pdf]

Supplementary Materials

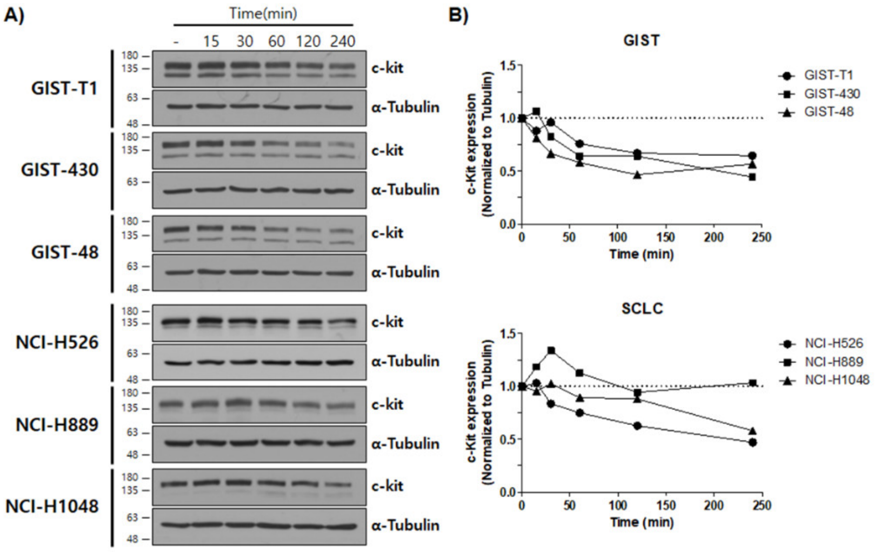

Supplementary Figure S1. Stability assessment of c-Kit protein. 4C9 antibody (10 μg/ml) was treated to each cell line with the indicated time point and the expression level of c-Kit was determined by western blot (A). (B) Total c-Kit protein was quantitated using ImageJ program and normalized to alpha-tubulin. The results represent the mean ± SD of three independent experiments. Alpha-tubulin was used as a loading control.

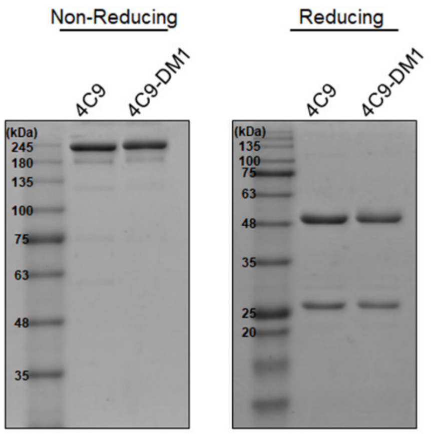

Supplementary Figure S2. Comparison of 4C9 and 4C9-DM1 in SDS-PAGE. Each 10 μg protein was loaded to SDS-PAGE and stained with Coomassie Brilliant Blue G250 and destained with 50% methanol.

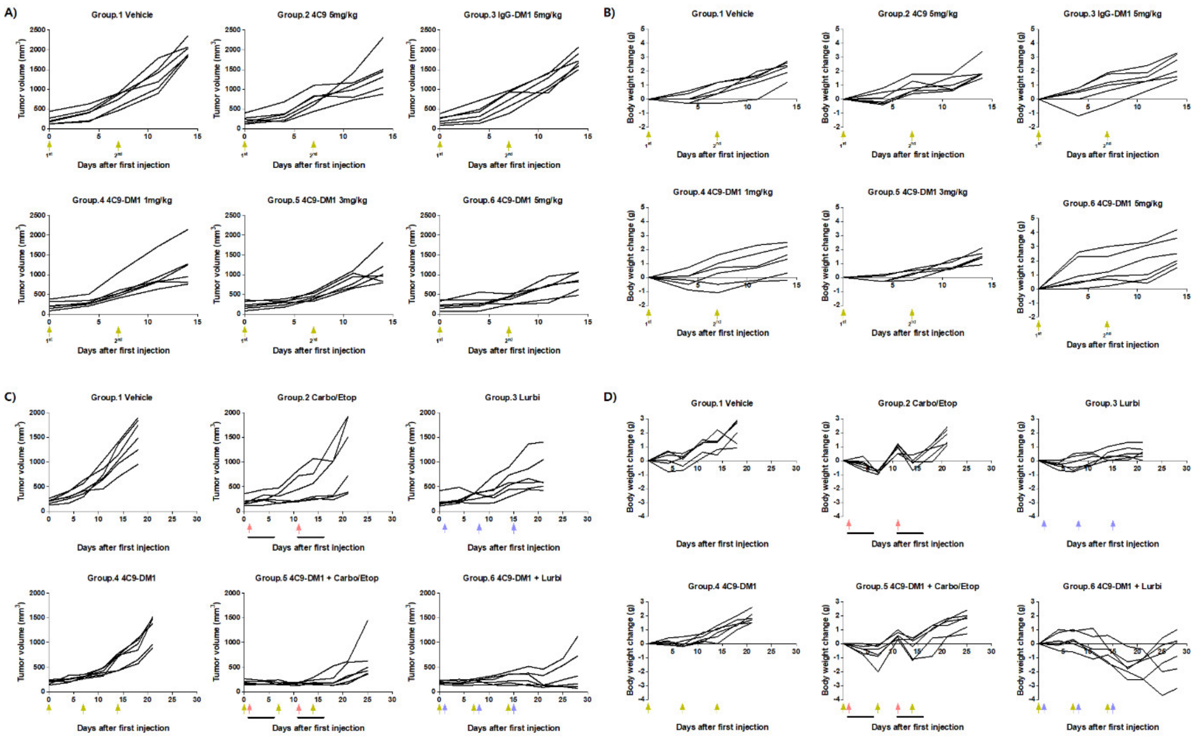

Supplementary Figure S3. Individual analysis of anti-tumor activity and body weight change. The results shown in figure 5 are presented for each individual (n = 6).

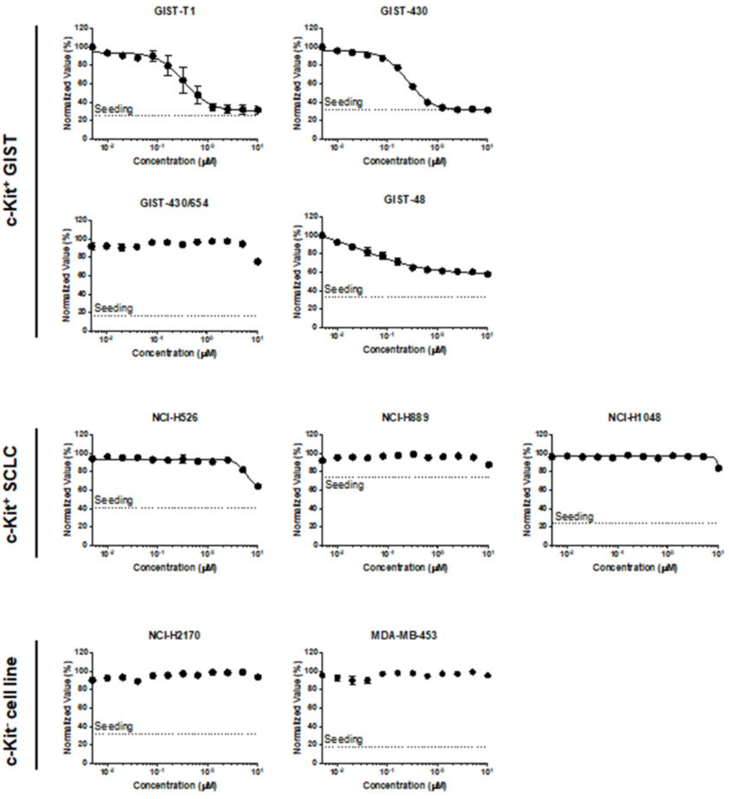

Supplementary Figure S4. The assessment of inhibitory concentration of imatinib in various cancer cell

lines. Cells were seeded into 96-well plates and incubated with imatinib for 3-4 days. Then, total cells were stained with Hoechst 33342 (10 μM) at 37 °C for 30 min and quantitated using a Celigo Imaging Cytometer. The results represent mean ± standard error of mean from at least three independent experiments.

Supplementary Table S1. *In vitro* IC_{50} value of imatinib

c-Kit expression	Tissue type	Cell line	Imatinib (μM)
c-Kit positive	GIST	GIST-T1	0.3357
		GIST-430	0.2497
		GIST-430/654[†]	N/E*
		GIST-48	0.0306
	SCLC	NCI-H526	5.924
		NCI-H889	N/E
		NCI-H1048	N/E
c-Kit negative	SCLC	NCI-H2170	N/E
	Breast cancer	MDA-MB-453	N/E

*N/E: not effective up to 10 Mm; †, this cell line is resistant to imatinib due to c-Kit activating mutation
